# Characteristics of Gut Microbiota in Patients with GH-Secreting Pituitary Adenoma

**DOI:** 10.1128/spectrum.00425-21

**Published:** 2022-01-12

**Authors:** Ben Lin, Meng Wang, Renyuan Gao, Zhao Ye, Yifei Yu, Wenqiang He, Nidan Qiao, Zengyi Ma, Chenxing Ji, Chengzhang Shi, Xiang Zhou, Yi Wang, Fangfang Zeng, Li Zhang, Wei Gong, Zhan Cao, Peng Zhou, Vladimir Melnikov, Hongying Ye, Yiming Li, Zhaoyun Zhang, Min He, Huanlong Qin, Yichao Zhang

**Affiliations:** a Department of Neurosurgery, Huashan Hospital, Shanghai Medical College, Fudan University, Shanghai, China; b Department of Endocrinology and Metabolism, Huashan Hospital, Shanghai Medical College, Fudan University, Shanghai, China; c Diagnostic and Treatment Center for Refractory Diseases of Abdomen Surgery, Shanghai Tenth People’s Hospital, Tongji University School of Medicine, Shanghai, China; d Shanghai Medical College, Fudan University, Shanghai, China; e National Center for Neurological Disorders, Shanghai, China; f Shanghai Key Laboratory of Brain Function and Restoration and Neural Regeneration, Shanghai, China; g Neurosurgical Institute of Fudan University, Fudan University, Shanghai, China; h Shanghai Clinical Medical Center of Neurosurgery, Shanghai, China; Emory University

**Keywords:** clinical therapeutics, metabolism, metagenomics

## Abstract

Prior study has demonstrated that gut microbiota at the genus level is significantly altered in patients with growth hormone (GH)-secreting pituitary adenoma (GHPA). Yet, no studies exist describing the state of gut microbiota at species level in GHPA. We performed a study using 16S rRNA amplicon sequencing in a cohort of patients with GH-secreting pituitary adenoma (GHPA, *n* = 28) and healthy controls (*n* = 67). Among them, 9 patients and 10 healthy controls were randomly chosen and enrolled in metagenomics shotgun sequencing, generating 280,426,512 reads after aligning to NCBI GenBank DataBase to acquire taxa information at the species level. Weighted UniFrac analysis revealed that microbial diversity was notably decreased in patients with GHPA, consistent with a previous study. With 16S rRNA sequencing, after correction for false-discovery rate (FDR), rank-sum test at the genus level revealed that the relative abundance of *Oscillibacter* and *Enterobacter* was remarkably increased in patients and *Blautia* and *Romboutsia* genera predominated in the controls, augmented by additional LEfSe (linear discriminant analysis effect size) analysis. As for further comparison at the species level with metagenomics sequencing, rank-sum test together with LEfSe analysis confirmed the enrichment of Alistipes shahii and Odoribacter splanchnicus in the patient group. Notably, LEfSe analysis with metagenomics also demonstrated that *Enterobacter* sp. *DC1* and *Enterobacter* sp. *940 PEND*, derived from *Enterobacter*, were both significantly enriched in patients. Functional analysis showed that amino acid metabolism pathway was remarkably enriched in GHPA, while carbohydrate metabolism pathway was notably enriched in controls. Further, significant positive correlations were observed between *Enterobacter* and baseline insulin-like growth factor 1 (IGF-1), indicating that *Enterobacter* may be strongly associated with GH/IGF-1 axis in GHPA. Our data extend our insight into the GHPA microbiome, which may shed further light on GHPA pathogenesis and facilitate the exploration of novel therapeutic targets based on microbiota manipulation.

**IMPORTANCE** Dysbiosis of gut microbiota is associated not only with intestinal disorders but also with numerous extraintestinal diseases. Growth hormone-secreting pituitary adenoma (GHPA) is an insidious disease with persistent hypersecretion of GH and IGF-1, causing increased morbidity and mortality. Researches have reported that the GH/IGF-1 axis exerts its own influence on the intestinal microflora. Here, the results showed that compared with healthy controls, GHPA patients not only decreased the alpha diversity of the intestinal flora but also significantly changed their beta diversity. Further, metagenomics shotgun sequencing in the present study exhibited that *Enterobacter* sp. *DC1* and *Enterobacter* sp. *940 PEND* were enriched in patients. Also, we were pleasantly surprised to find that the *Enterobacter* genus was strongly positively correlated with baseline IGF-1 levels. Collectively, our work provides the first glimpse of the dysbiosis of the gut microbiota at species level, providing a better understanding of the pathophysiological process of GHPA.

## INTRODUCTION

Trillions of microorganisms, i.e. microbiota, inhabit the gut and have coevolved to forge mutually beneficial relationships with the host for millions of years. The gut microbiome, which is contributed by microbiota, is viewed as an extracorporeal organ that communicates with and influences the host in unprecedented ways (1, 2). With the development of culture-independent molecular assays, especially 16S rRNA sequencing and metagenomics shotgun sequencing, the enormous diversity, functional capacity, and physiological dynamics of the gut microbiome have been elucidated (3, 4). Gut microbiota and a subset of their metabolites have been implicated in numerous physiological functions, including the intestinal barrier, host immune system, and glucose and lipid metabolism (1, 5). Conversely, alteration in the composition and function of gut microbiota, namely, gut microbiota dysbiosis, are involved in many diseases, such as cardiovascular disease, Parkinson’s disease, obesity, and type 1 diabetes (6–8).

The growth hormone (GH)/insulin-like growth factor 1 (IGF-1) axis plays a vital role in regulating intestinal homeostasis in an individual (9). Both clinical trials and animal experimentation have demonstrated that exogenous recombinant human GH (rhGH) administration improves macronutrient absorption and decreases intestinal damage and bacterial translocation under the condition of short bowel syndrome (10–13). Using an intestinal epithelial-specific GH receptor knockout mouse model, Young et al. verified that disruption of GH affects the intestinal gross anatomy, morphology, and function (14). Treatment with rhGH has also reportedly been used subcutaneously daily in patients with Crohn’s disease (15). More recently, GH has been associated with the alteration of the gut microbiome in adult male mice (16). As for the role of IGF-1 in gastrointestinal tract, previous studies demonstrated that IGF-1 regulates the proliferation, survival, and apoptosis of the epithelial cells (17). IGF-1 influences the presence of certain microbes in intestinal epithelial cells-specific IGF-1-knockout mice (18, 19). Intriguingly, germ-free mice are resistant to GH accompanied with low serum IGF-1 concentration, while microbial colonization has been shown to substantially increase IGF-1 levels (2, 20). These studies demonstrate the complexity of GH/IGF-1 axis in the gut and, unambiguously, suggest a potential mutual interplay between the gut microbiota and the GH/IGF-1 axis. However, data about the interaction between GH/IGF-1 axis and microbiota in humans are rare.

GH-secreting pituitary adenoma (GHPA), a common functional subtype of pituitary adenomas, is characterized by exceeding release of GH and IGF-1 (21). As a rare endocrinopathy, GHPA results in somatic overgrowth, i.e., acromegaly, and multiple systemic comorbidities, including diabetes, hypertension, and increased risk of colonic polyps and cancer (22–28). The latest research from Hacioglu et al. preliminary uncovered the altered composition of gut microbiota in seven patients with newly diagnosed acromegaly through 16S-based techniques (29). However, both a larger cohort and in-depth investigation are needed to unveil the causative relationship between acromegaly and microbial alteration.

## RESULTS

### Clinical characteristics of the participants.

In total, GH patients (*n* = 28) and healthy controls (*n* = 67) were enrolled in this study, with age and body mass index (BMI) adjusted. In patients with GHPA, 5 of 28 were suffering from recurrent GHPA, while the others were newly diagnosed. Levels of nadir GH and IGF-1 were 14.40 (5.12 to 22.47) ng/mL and 758.93 ± 146.10 ug/L, respectively. Clinical and biochemical findings of GHPA and healthy controls are summarized in Table 1, and the details of both groups are provided in Table S1 and S2, respectively.

**TABLE 1:** Baseline information of GH and HC groups^*a*^

Characteristic	Value for group		*P* value
	GH	HC	
Age (yrs)	38.18 ± 11.88	48.22 ± 2.96	<0.001
Gender			
Female	13 (46.4%)	39 (58.2%)	0.293
Male	15 (53.6%)	28 (41.8%)	
BMI (kg/m^2^)	25.87 ± 2.95	23.44 ± 2.94	0.001
HBP			
No	22 (78.6%)	59 (88.1%)	0.234
Yes	6 (21.4%)	8 (11.9%)	
DM			
No	25 (89.3%)	64 (95.5%)	0.255
Yes	3 (10.7%)	3 (4.5%)	
Recurrence			
No	23 (82.1%)		
Yes	5 (17.9%)		
Nadir GH (ng/mL)	14.40 (5.12–22.47)	NA	NA
IGF-1 (μg/L)	758.93 ± 146.10	NA	NA

a BMI: body mass index; HBP: hypertension; DM: diabetes mellitus; nadir GH: nadir level of growth hormone during OGTT test; recurrence: patient carried recurrent tumor despite past treatment; quantitative data are presented as mean ± standard deviation or median with interquartile range; categorical data are presented as frequency and percentage.

Clinical parameters, including BMI, fasting plasma glucose (FPG), fasting GH, nadir GH, IGF-1, and IGF-1 index, were compared preoperation and 6 months after trans-sphenoid endoscopic assisted surgery, the mainstay of GHPA treatment (Table 2). In our medical center, we use antibiotics after surgery rather than preoperatively for this surgery, and we collected fecal samples before surgery, therefore avoiding influence on the microbiota profile. In general, except for BMI (25.87 ± 2.95 kg/m^2^ versus 25.51 ± 3.04 kg/m^2^, *P* = 0.23), significant decrease was observed in clinical parameters, including FPG (5.26 ± 0.54 mmol/L versus 4.89 ± 0.49 mmol/L, *P* < 0.001), fasting GH (20.57 ± 15.55 ng/mL versus 2.17 ± 3.62 ng/mL, *P* < 0.001), nadir GH (16.23 ± 13.33 ng/mL versus 1.41 ± 2.33 ng/mL, *P* < 0.001), IGF-1 (758.93 ± 146.10 μg/L versus 369.42 ± 195.17 μg/lL, *P* < 0.001), and IGF-1 index (2.44 ± 0.74 versus 1.16 ± 0.59, *P* < 0.001). Nineteen out of 28 patients reached biochemical remission, defined as an age-normalized serum IGF-1 value and a random GH of <1.0 ng/mL (30), and 20 out of 28 patients achieved radiological remission, defined as no recurrence or residual tumor on follow-up magnetic resonance imaging.

**TABLE 2:** Comparison of clinical parameters before and after surgery^*a*^

Clinical parameter	Value from:		*P* value
	Preoperation	6 mo follow-up	
BMI (kg/m^2^)	25.87 ± 2.95	25.51 ± 3.04	0.23
FPG (mmol/L)	5.26 ± 0.54	4.89 ± 0.49	<0.001
Fasting GH (ng/mL)	20.57 ± 15.55	1.04 (0.32–2.44)	<0.001
Nadir GH (ng/mL)	14.40 (5.12–22.47)	0.43 (0.12–1.73)	<0.001
IGF-1 (ug/L)	758.93 ± 146.10	329.00 (214.75–428.50)	<0.001
IGF-1 index	2.44 ± 0.74	0.94 (0.75–1.34)	<0.001

a BMI: body mass index; FPG: fasting plasma glucose; fasting GH, nadir GH, and IGF-1index are defined in Materials and Methods.

### Altered microbial diversity in patients with GH-secreting pituitary adenoma.

**FIG 1:**
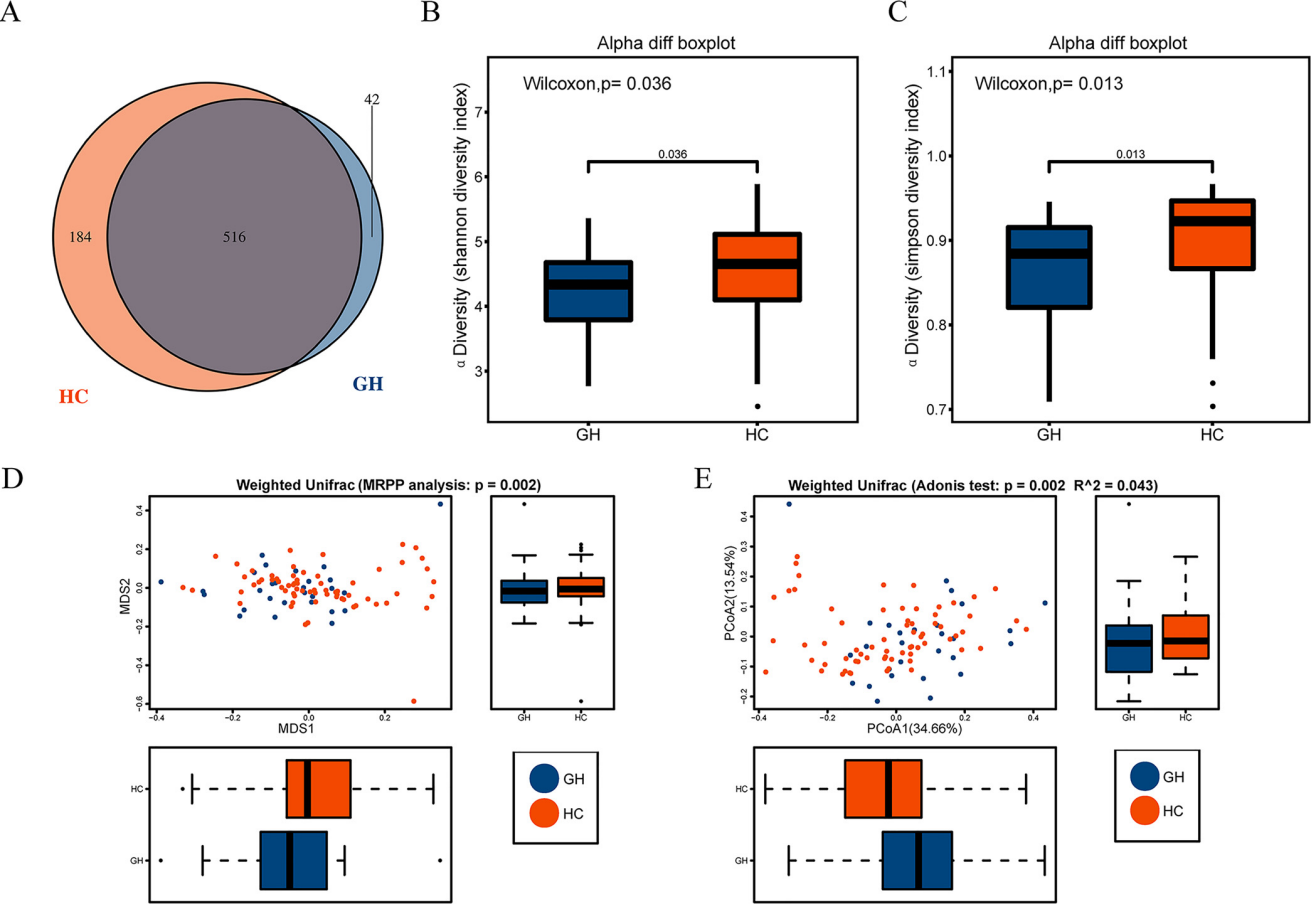
Microbial diversity was notably decreased in patients with GH-secreting pituitary adenoma. (A) A Venn diagram displaying the overlaps between groups showed that 516 of the total richness of 742 OTUs were shared among the two groups, while 42 and 184 OTUs were found exclusively in GH group and HC group, respectively. Compared with that in the controls, fecal microbial diversity, as estimated by the Shannon index (B) and Simpson index (C), was significantly decreased in patients with GH (*P* = 0.036 and 0.013). Beta diversity was calculated using weighted UniFrac and further analyzed by MRPP (D) and Adonis (E), both obtaining *P* values equal to 0.002. Further, the former and latter tests were presented via NMDS (D) and PCoA (E), respectively. Cooperatively, our results demonstrated a significant clustering tendency among all samples. GH, growth hormone-secreting pituitary adenoma; HC, healthy control; OTUs, operational taxonomy units; MRPP, multi response permutation procedure; NMDS, nonmetric multidimensional scaling; PCoA, principal coordinates analysis.

To investigate the microbial diversity between patients with GHPA and healthy controls (age and BMI adjusted), we characterized the clustering effects and found that 516 of the total 742 operational taxonomy units (OTUs) overlapped between patients with GHPA as GH group and healthy controls as HC group, while 42 OTUs were found specifically in GH group (Fig. 1A). Then, we analyzed the compositions of bacterial community between the two groups and detected the prominently altered gastrointestinal microbial diversity. Compared with that in the control group, fecal microbial diversity, assessed by Shannon index (Fig. 1B) and Simpson index (Fig. 1C), was notably decreased in GH group (*P* = 0.036 and *P* = 0.013, respectively). To demonstrate microbiome space between the groups, beta diversity was evaluated by weighted UniFrac, and intergroup variance was further presented via nonmetric multidimensional scaling (NMDS) along with *P* value acquired by multiresponse permutation procedure (MRPP) (Fig. 1D) and principal coordinates analysis (PCoA) along with Adonis analysis (Fig. 1E). Notable change was observed between two groups (*P* = 0.002 for MRPP analysis and *P* = 0.002 for Adonis test). Collectively, compared to that in the healthy controls, microbial diversity was significantly decreased in GH group, suggesting the potential vulnerability to various pathological conditions (31, 32).

### Taxonomic changes of gut microbiota at genus level in patients with GH-secreting pituitary adenoma detected by 16S rRNA sequencing.

**FIG 2:**
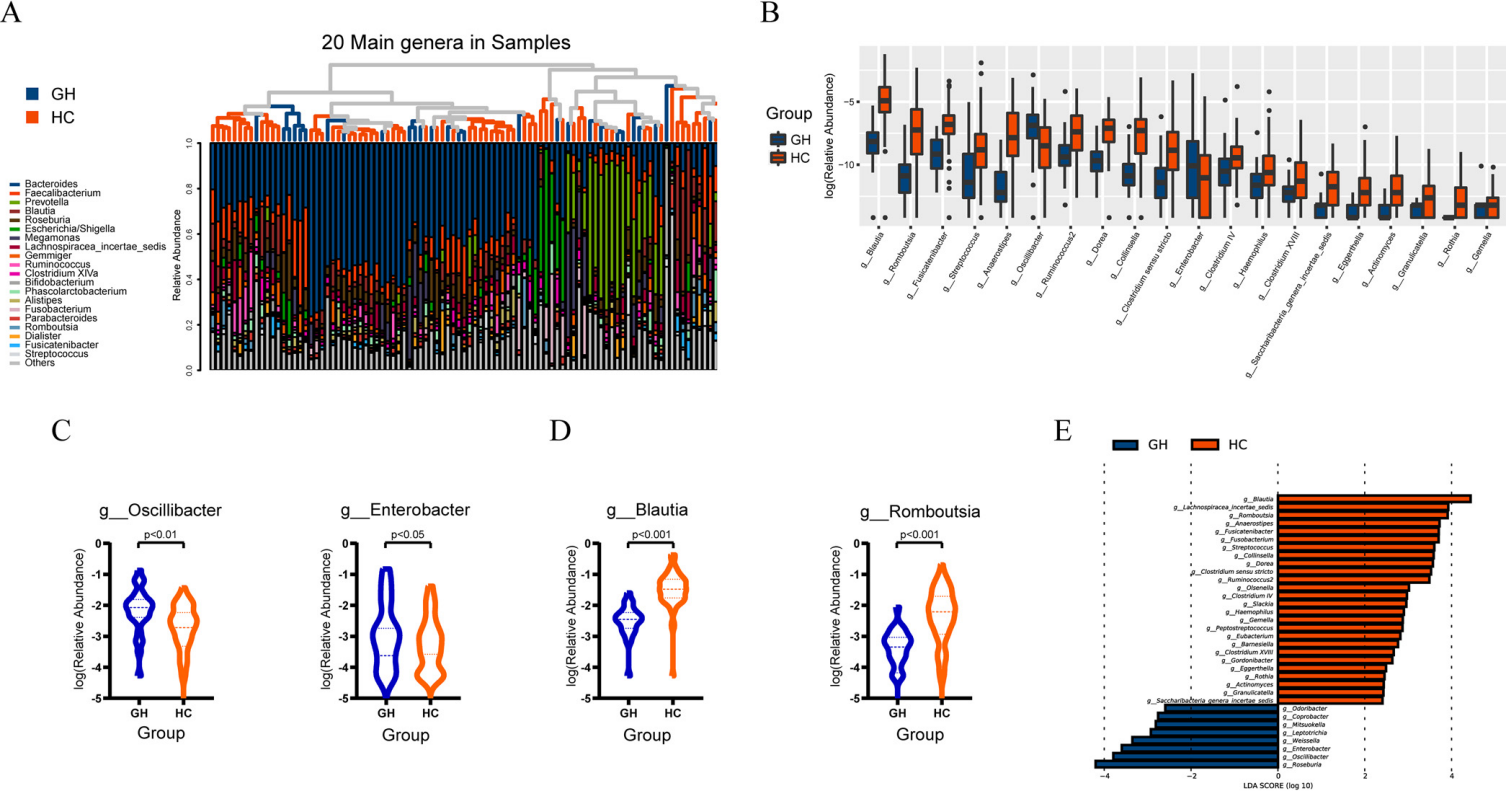
Taxonomic changes of gut microbiota at genus level in patients with GH-secreting pituitary adenoma. (A) A histogram displayed the relative abundance of the 20 main genera in all samples, simultaneously showing the clustering relationships among all samples. (B) A barplot presenting the phylogenetic profiles at genus level of the two groups, with only *Oscillibacter* genus and *Enterobacter* genus increased in GH group while *Blautia* genus and *Romboutsia* genus ranked top 2 among the decreased genera. (C) Two violin plots demonstrate significantly increased abundance of *Oscillibacter* genus and *Enterobacter* genus in GH group. (D) Two violin plots show significantly decreased abundance of *Blautia* genus and *Romboutsia* genus in GH group. (E) A histogram exhibits genera with significant effects on the division between groups, assessed by LEfSe analysis and corresponding influence represented as LDA score, further confirming the prominent roles of *Oscillibacter*, *Enterobacter*, *Blautia*, and *Romboutsia* genera. GH, growth hormone-secreting pituitary adenoma; HC, healthy control; g_ in figure c and d is short for genus, dotted line and dashed line represent quartiles and median, respectively; LDA, linear discriminant analysis.

The signature sequence from OTUs was then aligned to the database of known species (Ribosomal Database Project database) and annotated with ranges of taxonomic levels. As shown in Fig. 2A, *Bacteroides*, *Faecalibacterium*, and *Prevotella* were the dominant genera in all fecal samples. Total composition of bacterial community at the genus level in all participants is presented in Fig. S2A in the supplemental material. In addition, the relative abundance of phyla *Bacteroidetes* and *Firmicutes* and the corresponding *Firmicutes/Bacteroidetes* ratio were illustrated in Fig. S2B, indicating an increased abundance of *Bacteroidetes* along with decreased *Firmicutes/Bacteroidetes* ratio, consistent with previous research.

In order to identify specific bacterial taxa associated with GHPA, we compared fecal microbiota by means of Wilcoxon rank-sum test with false-discovery rate (FDR) correction and LEfSe analysis and found several differential taxa at genus level between GH group and HC group (age and BMI adjusted). Strikingly, compared to that in the control group, abundance of only two taxa, *Oscillibacter* (FDR-adjusted *P* = 0.003) and Enterobacter genus (FDR-adjusted *P* = 0.043), was substantially increased in GH group (Fig. 2B and C), while the remaining genera were all significantly decreased in GH group, and *Blautia* (FDR-adjusted *P* < 0.001) and *Romboutsia* genera (FDR-adjusted *P* < 0.001) ranked in the top 2 (Fig. 2B and D). The results of LEfSe analysis, sorting taxa according to results of Kruskal-Wallis test, confirmed the prominent roles of *Oscillibacter* (*P* < 0.001), *Enterobacter* (*P* = 0.007), *Blautia* (*P* < 0.001), and *Romboutsia* (*P* < 0.001), with linear discriminant analysis (LDA) scores of 3.79, 3.59, 4.44, and 3.91, respectively (Fig. 2E). According to existing reports, the increase of *Oscillibacter* is associated with inflammatory status as well as increased permeability of the gastrointestinal tract (33–37). Meanwhile, in addition to acting as an opportunistic pathogen (38), the expanded *Enterobacter* may reinforce the fermentation and utilization of various carbon sources to produce metabolites such as acetic acid (39–41). On the contrary, the general reduction of other genera, consistent with decreased biodiversity, might confront patients with susceptibility to opportunistic pathogens (42). The reduction of gastrointestinal microbial diversity has frequently been related to various diseases, including diabetes, dyslipidemia, obesity, and hypertension (32, 43, 44). Previous studies have proposed that decreased gastrointestinal microbial diversity is frequently associated with a bloom of commensals that may become detrimental (pathobionts) and contribute to systemic inflammation (42, 45). As growth hormone (GH) and insulin-like growth factor-1 (IGF-1) play important roles in regulating the body metabolism, patients with growth hormone-secreting pituitary adenoma (GHPA) present with multiple metabolic abnormalities, such as diabetes (20 to 56%) and hyperlipidemia (13 to 51%) (22–24). In the present study, our data showed that gut microbial diversity in patients with GHPA was significantly decreased, which was similar to that found in patients with diabetes and dyslipidemia, and we therefore suspect that decreased microbial diversity might intermediate the vulnerability of GHPA patients to metabolic disturbances.

### Taxonomic differences at species level between patients with GH-secreting pituitary adenoma and healthy controls.

**FIG 3:**
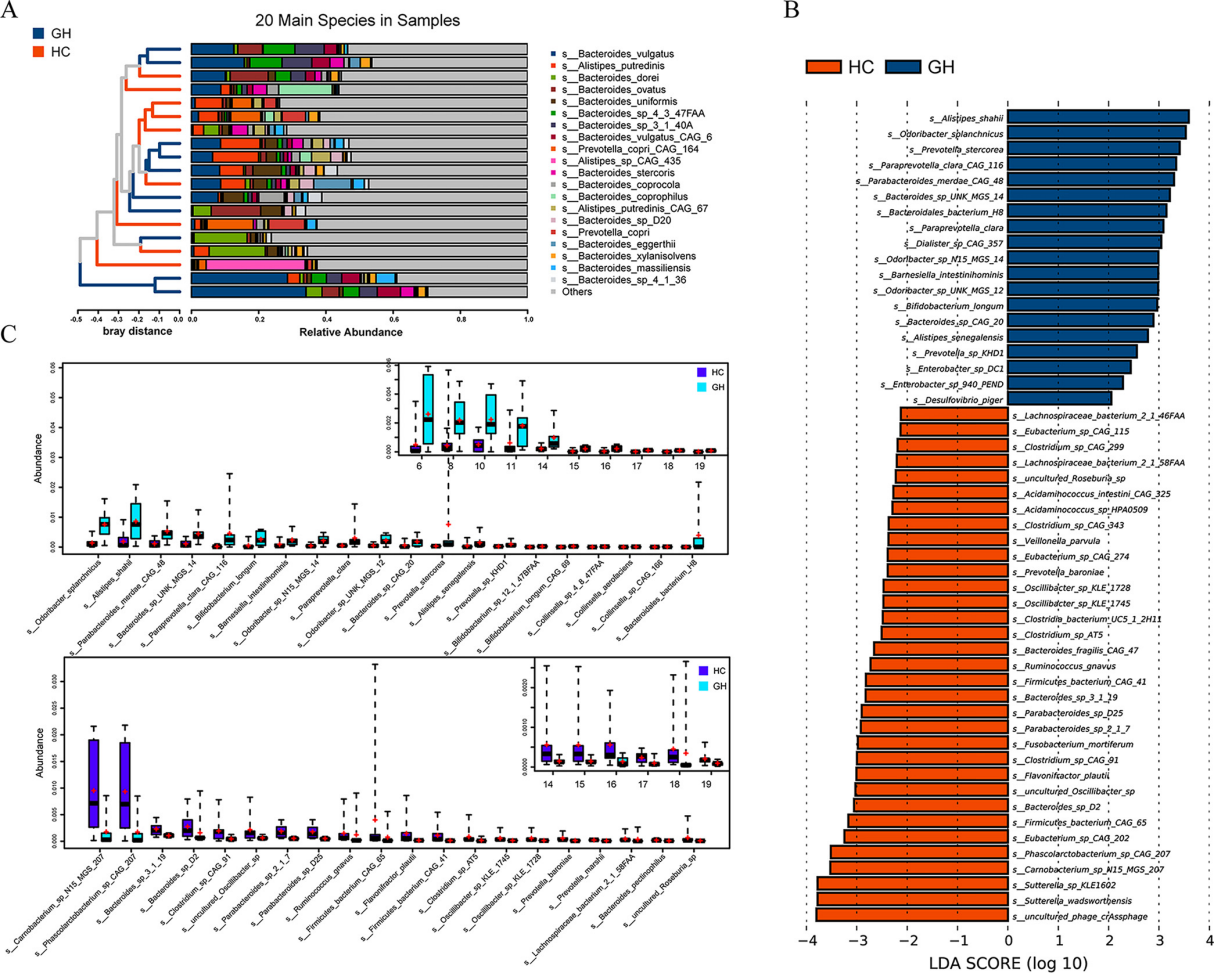
Taxonomic differences at species level between patients with GH-secreting pituitary adenoma and healthy controls. (A) A histogram shows the relative abundance of the 20 main species in all samples, with a clustering tree of the total samples listed on the left side and the specific species exhibited on the right side. (B) The histogram demonstrates species with absolute LDA score exceeding two, identifying species with significant influence on sample division. (C) Boxplots illustrate differential phyla at species level in patients and healthy individuals detected by rank-sum test, with two different colors representing different sets of samples, while the short bar and the plus sign indicate the median and the mean, respectively. Only the 20 most abundant species in each group are shown for clarity. GH, growth hormone-secreting pituitary adenoma; HC, healthy control; LDA, linear discriminant analysis.

To further illuminate the comprehensive landscape of taxonomic alteration at species level in GHPA, fecal samples of 9 patients with GHPA along with those of 10 participants from HC group were randomly selected and analyzed by metagenomics shotgun sequencing (age and BMI adjusted). The metagenome results data are presented in Table S3. Profiles of phylotype at species level were presented, and Bacteroides vulgatus, Alistipes putredinis, Bacteroides dorei, and Bacteroides ovatus were the top four species (Fig. 3A). LEfSe analysis, taxa stratified according to Kruskal-Wallis test, showed that Alistipes shahii (*P* = 0.041, LDA score = 3.59) and Odoribacter splanchnicus (*P* = 0.007, LDA score = 3.53), together with Prevotella stercorea (*P* = 0.002, LDA score = 3.41), were notably enriched in the GH group, while uncultured phage crAssphage (*P* = 0.002, LDA score = 3.80), Sutterella wadsworthensis (*P* = 0.022, LDA score = 3.78), and *Sutterella* sp. *KLE1602* (*P* = 0.007, LDA score = 3.78) were strikingly enriched in the healthy control group (Fig. 3B). It is worth noting that species *Enterobacter* sp. *DC1* (*P* = 0.041, LDA score = 2.44) and *Enterobacter* sp. *940 PEND* (*P* = 0.014, LDA score = 2.28) that were derived from *Enterobacter* were both prominently enriched in the GH group (Fig. 3B). Results of rank-sum test demonstrated that certain species, including Odoribacter splanchnicus (*P* = 0.006) and Alistipes shahii (*P* = 0.043), were remarkably increased in the GH group, while species such as *Carnobacterium* sp. *N15 MGS 207* (*P* = 0.028), *Phascolarctobacterium* sp. *CAG 207* (*P* = 0.030), and *Bacteroides* sp. *3_1_19* (*P* = 0.013) were notably increased in the HC group (Fig. 3C). Since the numbers of participants enrolled in 16S and metagenomics sequencing were unparalleled, variations in taxonomic profiles were inevitable, and rank-sum test at genus level (Fig. S2D) with metagenomics has revealed deviation from 16S rRNA sequencing with respect to differential taxa between GH and HC groups, thus causing difficulties in alignment and interpretation of results. For example, genus *Oscillibacter* was enriched in the GH group in the 16S study while the abundance of species derived from this genus was decreased in the GH group with metagenomics, probably resulting from systemic bias due to unequal numbers of participants. Comparison of notably altered taxa at genus level as well as the abundance of *Oscillibacter* with metagenomics (Fig. S2C) confirmed the inconsistency in regard to relative abundance of genus *Oscillibacter* between 16S (Fig. 2C) and metagenomics sequencing (Fig. S2C). Yet, despite the potential shift in taxonomic construction, results of *Enterobacter* at both genus and species levels were compatible, further augmenting its significance in GH group.

### Functional characterization of microbiome in GH patients.

Next, we set out to probe for the functional profiles and the differences in the metabolic potential of gut microbiota in GHPA and healthy controls. Together, a noteworthy proportion of the overall genes in both groups were involved in metabolism, genetic information processing, and environmental information processing (Fig. S3A), corroborated by depiction of relative abundance of comprehensive elements in each group at level 1 (Fig. S3B) and level 2 (Fig. S3C).

Kyoto Encyclopedia of Genes and Genomes (KEGG) orthologs (KOs) is a taxonomic system of proteins (enzymes), in which proteins with highly similar sequences and similar functions along the same pathway are grouped together (46). To depict the detailed landscape of differential functional modules between the GH group and the control group, we initiated further comparison of KOs between the two groups by means of rank-sum test as well as LEfSe analysis and further annotated the varied KOs to related pathways.

**FIG 4:**
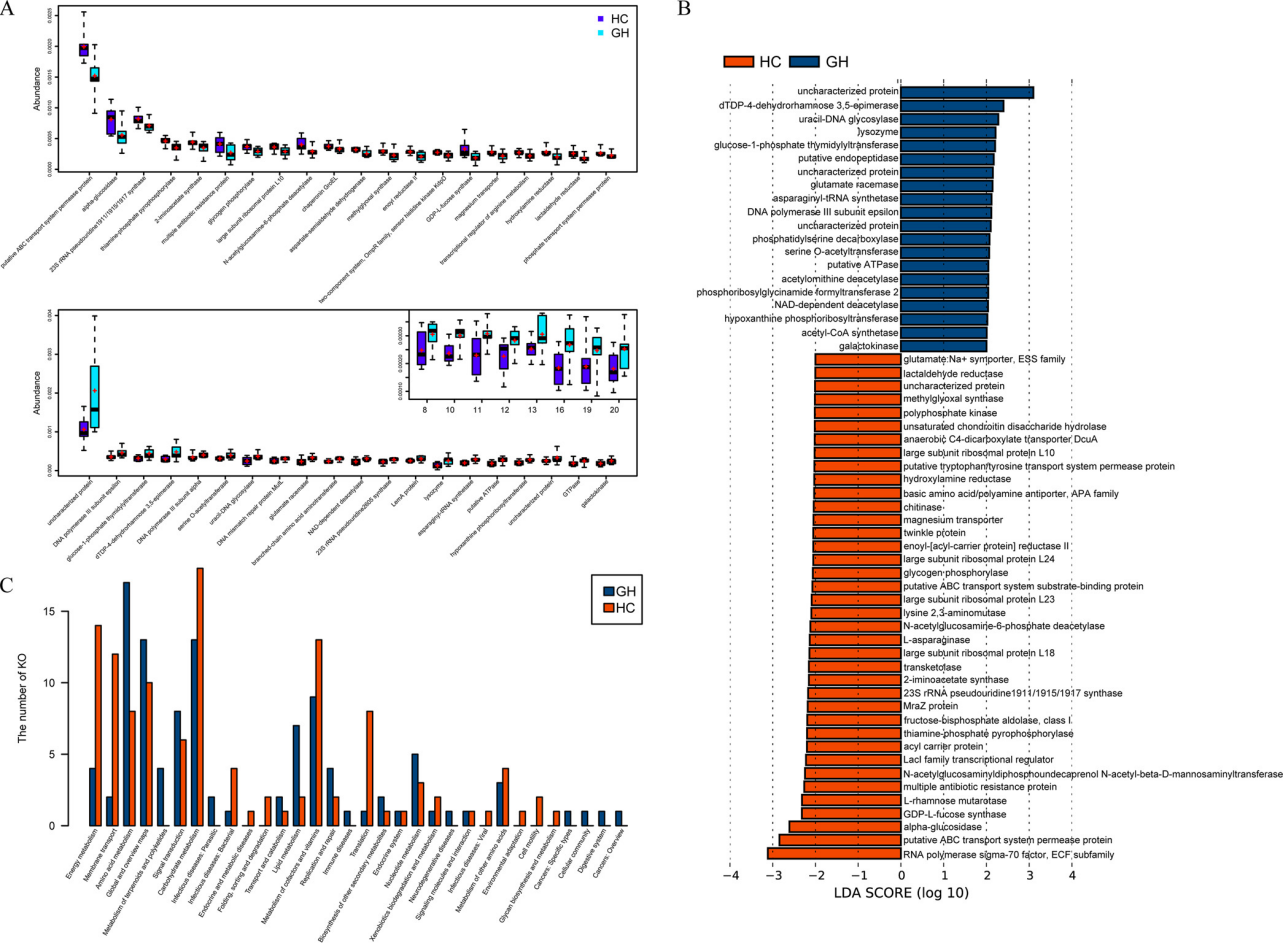
Functional characterization of microbiome in GH patients. (A) Boxplots illustrate differential KOs between GH group and HC group detected by rank-sum test, with two different colors representing different sets of samples while the short bar and the plus sign indicating the median and the mean, respectively. Only the 20 most abundant KOs in each group are shown for clarity. (B) The histogram shows KOs with absolute LDA score exceeding two, identifying KOs with significant influence on sample division. (C) The barplot displays the percentage of the overall KOs occupied by each pathway in both groups, respectively, with amino acid metabolism pathway notably enriched in GHPA while carbohydrate metabolism pathway remarkably enriched in controls. GH, growth hormone-secreting pituitary adenoma; HC, healthy control; LDA, linear discriminant analysis; KO, KEGG ortholog.

According to the KOs abundance table, the *P* value of KOs’ difference between the two groups was calculated by rank-sum test. KOs with significantly different expression between the experimental group and the control group were screened according to the *P* value of <0.05. As a result, putative ABC transport system permease protein (*P* = 0.002), alpha-glucosidase (*P* = 0.028), 23S rRNA pseudouridine1911/1915/1917 synthase (*P* = 0.028), and thiamine-phosphate pyrophosphorylase (*P* = 0.004) were proved to be the top 4 abundant KOs in HC group (Fig. 4A), while an uncharacterized protein (*P* = 0.017), DNA polymerase III subunit epsilon (*P* = 0.017), glucose-1-phosphate thymidylyltransferase (*P* = 0.035), and dTDP-4-dehydrorhamnose 3,5-epimerase (*P* = 0.043) were the 4 KOs with the highest abundance in the GH group (Fig. 4A).

As for LEfSe analysis, linear discriminant analysis (LDA) was used to estimate the effects on sample classification resulting from each KO. In the GH group, uncharacterized protein (*P* = 0.022, LDA score = 3.79), dTDP-4-dehydrorhamnose 3,5-epimerase (*P* = 0.027, LDA score = 3.15), uracil-DNA glycosylase (*P* = 0.011, LDA score = 3.03), and lysozyme (*P* = 0.022, LDA score = 2.87) were the most significantly enriched KOs, with RNA polymerase sigma-70 factor, the extracytoplasmic function (ECF) subfamily (*P* = 0.041, LDA score = 4.09), putative ABC transport system permease protein (*P* = 0.004, LDA score = 3.77), alpha-glucosidase (*P* = 0.014, LDA score = 3.38), and GDP-l-fucose synthase (*P* = 0.034, LDA score = 2.99) notably enriched in the counterpart (Fig. 4B). According to the relative abundance of each KO from the two groups, we further annotated the detected KOs to the existent pathways in KEGG database, acquiring the percentage of the overall KOs occupied by each pathway. Amino acid metabolism (17 in GH versus 8 in HC) together with global and overview maps (13 in GH versus 10 in HC) were the most remarkably enriched pathways in the GH group. In contrast, energy metabolism (4 in GH versus 14 in HC), membrane transport (2 in GH versus 12 in HC), and carbohydrate metabolism (13 in GH versus 18 in HC) were the most notably enriched pathways in the HC group (Fig. 4C).

Cooperatively, rank-sum test and LEfSe analysis illustrated the enrichment of KOs related to glycan metabolism (dTDP-4-dehydrorhamnose 3,5-epimerase and glucose-1-phosphate thymidylyltransferase), amino acid metabolism (putative endopeptidase, glutamate racemase, asparaginyl-tRNA synthetase, serine O-acetyltransferase, acetylornithine deacetylase, and phosphoribosylglycinamide formyltransferase 2), and utilization of certain carbohydrates (acetyl-CoA synthetase and galactokinase), in spite of the overall decline in KOs associated with carbohydrate metabolism. As reported by existed researches, amino acid metabolism and carbohydrate metabolism are involved in the production of short-chain fatty acids (SCFA) (47), while glycan metabolism is related to pathogen recognition and inflammation (48, 49). Thus, the functional profiles of gastrointestinal microbiota in GH patients may indicate the variance in SCFA metabolism and inflammatory status.

To capture the driving factors potentially causing the functional changes and respective contribution, FishTaco analysis was initiated (50). According to our results, Alistipes shahii was a major contributor with respect to amino acids metabolism, carbohydrate metabolism, and energy metabolism, while species of *Enterobacter* were the suppressive factors in amino acids metabolism, carbohydrate metabolism, and energy metabolism (Fig. S4). Though the FishTaco analysis outlined the prominence of GH-enriched taxa in respect to functional change, the results were not fully consonant with regard to the role of *Enterobacter*, defined as a suppressive factor in amino acids metabolism. Given the limitations of FishTaco analysis, further exploration into the exact role of this taxon is warranted.

### Correlation analysis between the differential taxa and clinical indices of patients with GH-secreting pituitary adenoma.

In order to uncover the potential relationship between gastrointestinal microbiota and clinical characteristics of GH-secreting pituitary adenoma, we initiated spearman correlation test between differential taxa at the genus level and the species level and the clinical index, including Knosp grade, tumor size, granulation extent of adenoma, radiological remission, biochemical remission, preoperative (pre-op) nadir GH, change in nadir GH, preoperative (pre-op) fasting GH, change in fasting GH, preoperative (pre-op) IGF-1 index, preoperative (pre-op) IGF-1, preoperative (pre-op) FPG, change in IGF-1 index, ratio of change in IGF-1 index, ratio of change in fasting GH, and ratio of change in nadir GH.

**FIG 5:**
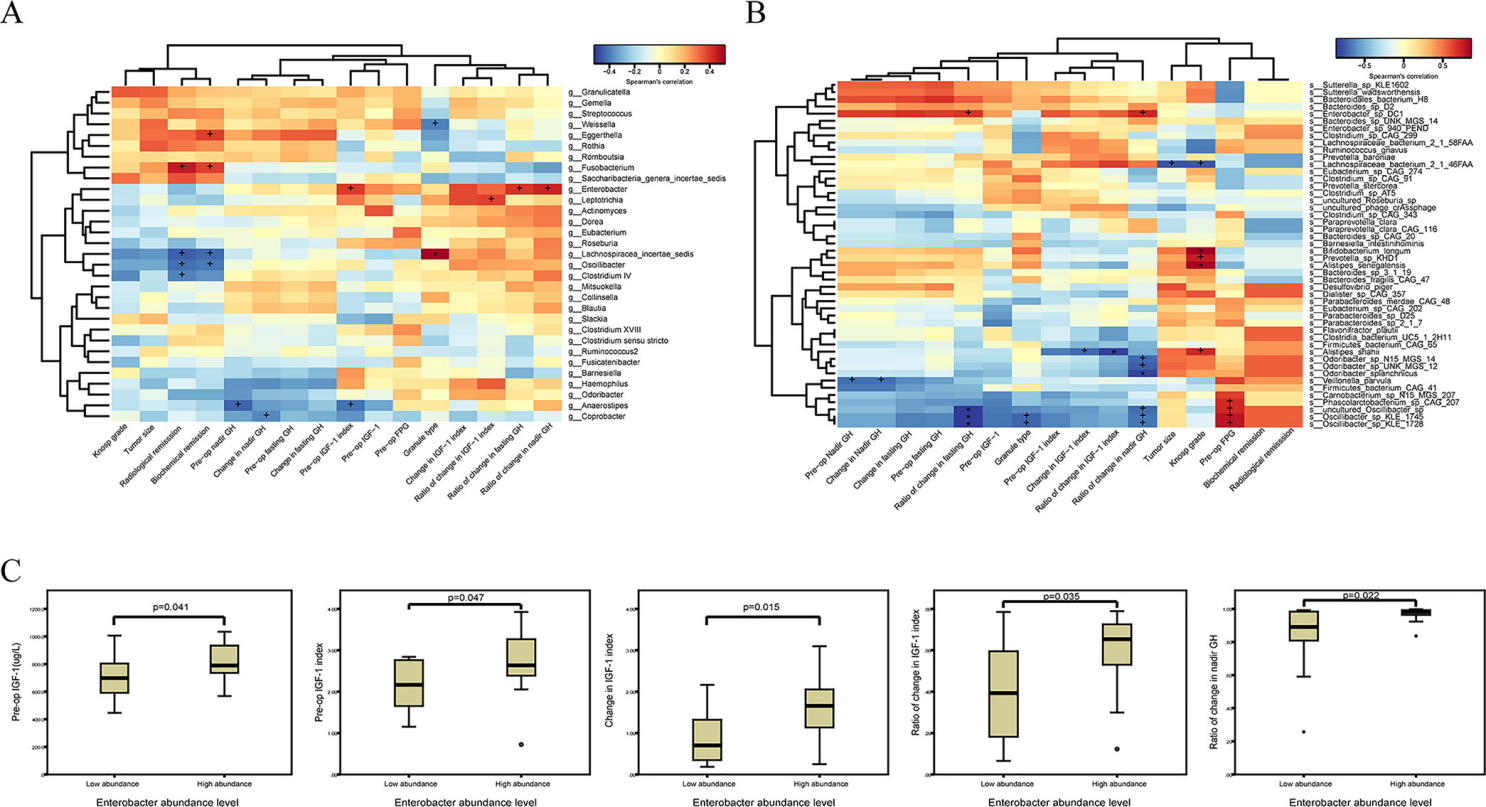
Correlation analysis between the differential taxa and clinical indices of patients with GH-secreting pituitary adenoma. (A) The heatmap delineates the correlation between significantly varied genera detected by LEfSe analysis and clinical indices of participants enrolled in 16S rRNA sequencing after spearman correlation analysis, among which prominent positive correlation was observed between *Enterobacter* genus and pre-op IGF-1. (B) Similarly, spearman correlation analysis was conducted between remarkably differed species observed in LEfSe analysis and clinical parameters of patients registered in metagenomics sequencing. The depth of color directly shows the degree of correlation between taxon and environmental factors. At the same time, correlation significance test was carried out, with + and * symbolizing *P* < 0.05 and *P* < 0.01, respectively. (C) The boxplots demonstrate that significant differences existed in pre-op IGF-1, pre-op IGF-1 index, change in IGF-1 index, ratio of change in IGF-1 index, and ratio of change in nadir GH between low-abundance group and high-abundance group, divided according to relative abundance of *Enterobacter* genus in GH group. Detailed definitions of clinical indices involved in correlation analysis are illustrated in Materials and Methods.

At the genus level, notably, the *Fusobacterium* genus was positively correlated with both radiological remission (*r* = 0.46, *P* = 0.013) and biochemical remission (*r* = 0.39, *P* = 0.042), while *Lachnospiraceae incertae sedis*, another taxon enriched in the control group, was negatively correlated with radiological remission (*r* = −0.45, *P* = 0.016) and biochemical remission (*r* = −0.43, *P* = 0.022). Meanwhile, *Oscillibacter*, prominently enriched in the GH group as seen in Fig. 2B to E, was also negatively correlated with radiological remission (*r* = −0.42, *P* = 0.026) and biochemical remission (*r* = −0.40, *P* = 0.034) (Fig. 5A). Additionally, a significant positive correlation is detected between *Lachnospiraceae incertae sedis* and the densely granulated type of GH-secreting pituitary adenoma (*r* = 0.52, *P* = 0.005), which suggests the lower aggressiveness and higher response rate to pharmacological intervention compared to those of the sparsely granulated type (Fig. 5A). Furthermore, *Enterobacter*, which was enriched in GHPA as shown in Fig. 2B to E, was positively correlated with preoperation IGF-1 index (*r* = 0.39, *P* = 0.038), ratio of change in fasting GH (*r* = 0.41, *P* = 0.032), and ratio of change in nadir GH levels (*r* = 0.44, *P* = 0.018) (Fig. 5A).

At the species level, strikingly, *Enterobacter* sp. *DC1* was positively correlated with ratio of change in fasting GH (*r* = 0.68, *P* = 0.043) and ratio of change in nadir GH levels (*r* = 0.72, *P* = 0.028), in line with the correlation analyses of *Enterobacter*. With regard to preoperation IGF-1 index, *Enterobacter* sp. *DC1* was not significantly associated with this parameter in a positive manner (*r* = 0.58, *P* = 0.108), and this association was less remarkable than that observed between genus *Enterobacter* and preoperation IGF-1 index mentioned above (*r* = 0.39, *P* = 0.038). Collectively, correlation analyses of *Enterobacter* at genus and species levels revealed its potential fundamental role in GHPA. Meanwhile, species of *Odoribacter*, including Odoribacter splanchnicus (*r* = −0.82, *P* = 0.007), *Odoribacter* sp. *UNK MGS 12* (*r* = −0.75, *P* = 0.020), and *Odoribacter* sp. *N15 MGS 14* (*r* = −0.72, *P* = 0.030), were inversely correlated with ratio of change in nadir GH. However, Alistipes shahii, enriched in GHPA, was negatively related to the change in IGF-1 index (*r* = −0.72, *P* = 0.037) and ratio of change in IGF-1 index (*r* = −0.83, *P* = 0.008) (Fig. 5B).

Based on the results above, we then focused on the *Enterobacter* genus to investigate its possible influence on clinical parameters. According to the relative abundance of the *Enterobacter* genus, we divided patients into two groups: patients with low abundance of *Enterobacter* (low-abundance group, *n* = 14) and patients with high abundance of *Enterobacter* (high-abundance group, *n* = 14). As shown in Fig. 5C, compared with those in the low-abundance group, preoperative IGF-1 (*P* = 0.041) and IGF-1 index (*P* = 0.047) were significantly higher in patients with high abundance of *Enterobacter*. In addition, ratio of change in IGF-1 index (*P* = 0.035) and ratio of change in nadir GH (*P* = 0.022) were also higher in patients with high abundance of *Enterobacter* than in the low-abundance group.

### Gut microbes discriminate patients with GH-secreting pituitary adenoma from healthy controls.

**FIG 6:**
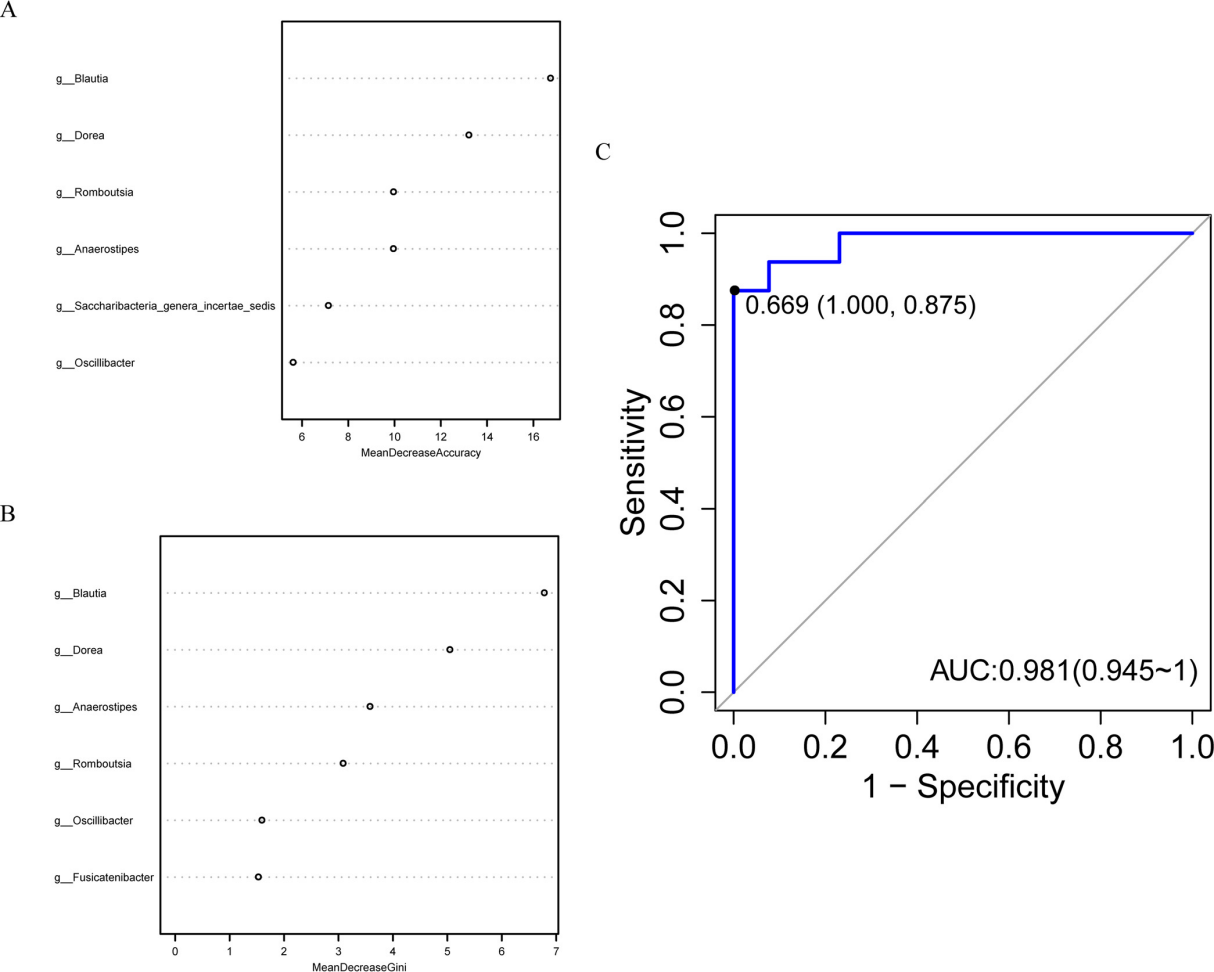
Gut microbes discriminate patients with GH-secreting pituitary adenoma from healthy controls. The scatter diagrams on the left side display the respective top 6 genera with largest contributions to the division of the two sample sets, corresponding to mean decrease accuracy (A) and mean decrease Gini (B) separately. (C) The ROC curve of the random forest model constructed on the basis of ordered differential genera, in which the abscissa axis is 1 − specificity while the ordinate is sensitivity. The point marked in the figure is the closest one to the top left corner, with a specificity and sensitivity equal to 1.000 and 0.875, respectively. AUC, the area under the curve, has reached 0.981 in our model, and the larger the AUC, the better the prediction effect of the model. g_ in panels A and B is an abbreviation of genus.

To investigate the latent role of gut microbiome as the biomarker of GHPA, we constructed a random forest classifier model which could discriminate the patients from healthy controls with better specificity and sensitivity. We first conducted a 10-fold cross-validation on a random forest model between the patients (*n* = 15) and healthy controls (*n* = 51). As a result, 6 genera were selected as the optimal markers and were ranked according to the relative degree of contribution as *Blautia*, *Dorea*, *Romboutsia*, *Anaerostipes*, *Saccharibacteria genera incertae sedis*, and *Oscillibacter* in regard to mean decrease accuracy (Fig. 6A), while genera *Blautia*, *Dorea*, *Anaerostipes*, *Romboutsia*, *Oscillibacter*, and *Fusicatenibacter* ranked in the top 6 with respect to mean decrease Gini (Fig. 6B). Finally, we utilized the remaining samples (samples of 13 GHPA and 16 healthy controls) to validate the diagnostic efficacy of this model. Area under the curve (AUC) value was 0.981, indicating the potential of gut microbiome as a biomarker for GHPA (Fig. 6C). This machine-learning algorithm calculated mean decrease accuracy along with mean decrease Gini to determine the order in which taxa were selected and evaluate the relative importance of individual taxa in respect to classification (GH group or control group). Nevertheless, this algorithm reflected relative importance of individual taxa with respect to classification, and it did not generate the result that these taxa were the most significantly varied taxa between the two groups. Given the fact that genus *Enterobacter* was not ranked top among the most notably altered taxa in 16S rank-sum test and LEfSe analysis, it may explain the reason why the specific taxon was not included in the parameters for robust differentiation. Collectively, our results demonstrate a substantial link between gastrointestinal microbiome and vital parameters of GH patients, hinting at the prospective role of microbiome in GHPA.

## DISCUSSION

Here, our results show a significantly altered microbial composition in patients with GH-secreting pituitary adenoma (GHPA). Compared to an earlier study investigating microbiota in GHPA patients using 16S-based techniques, the chosen methods allowed for the detection of changes at the species level (29). We confirmed a decreased diversity of microbiota in the patients compared with that in healthy controls and found increased levels of *Oscillibacter* and *Enterobacter* genera in GHPA and uncovered a strong association of *Enterobacter* with GH/IGF-1 axis in the disease.

High diversity in microbiota may protect the host against diseases (51, 52), and on the contrary, decreased diversity has been associated with various pathological conditions such as diabetes, dyslipidemia, obesity, and hypertension (32, 44, 53). A previous study including 7 patients with acromegaly observed a significantly less diverse microbiota in the patients than in controls by 16S rRNA amplicon sequencing (29). In our study, we confirmed the remarkably decreased diversity of microbiota in the patients compared to that in healthy controls, consistent with the prior study (29). Also, Hacioglu et al. demonstrated the increased abundance of phylum *Bacteroidetes* along with a decreased *Firmicutes/Bacteroidetes* ratio in the same study, and we found similar results in our study, strengthening its role as a characteristic change (Fig. S2B and C). At the genus level, Hacioglu et al. showed the decrease of genera *Bifidobacterium*, *Collinsella*, *Butyricimonas*, *Clostridium*, *Oscillospira*, and *Dialister*, along with the increase of genus *Bacteroides.* However, we detected a decrease of *Collinsella* while *Bifidobacterium*, *Bacteroides*, *Butyricimonas*, *Dialister*, and *Clostridium* were not significantly altered, with *Oscillospira* not detected. As for genera *Enterobacter* and *Oscillibacter*, both were not found among the notably differed taxa in the study of Hacioglu et al. With regard to the reason for the variance among the two study, we suspected that it was probably a consequence of divergent long-term dietary patterns between participants in two groups (54).

To the best of our knowledge, this is the first report to demonstrate the alteration of microbiota at the species level in GHPA. Work from Hacioglu et al. first provided a better understating for the gut microbiota in GHPA, while owing to the limitations, i.e., small number of patients and lack of the high-resolution taxonomic detection at lower clade levels, a more in-depth study was needed to detect altered gut microbiota at species level in acromegaly. Herein, we expanded the number of participants, uncovered the microbial composition at the species level, explored the functional characterization of GHPA microbiome, and demonstrated the correlation between microbiome and clinical indices of patients with GHPA.

In our results, we demonstrate that the abundance of *Blautia* genus and *Romboutsia* genus is lower, while *Oscillibacter* genus and *Enterobacter* genus levels are increased in fecal samples of patients with GHPA. *Blautia* genus has been reported to be significantly decreased in numerous diseases, including diabetes, obesity, cancers, and various inflammatory diseases (55, 56). *Romboutsia* is an anaerobic taxon first reported by Gerritsen et al. in 2014 (57). Milani et al. revealed that *Romboutsia* was significantly decreased in patients with Clostridium difficile infection, which might trigger the blooming of Clostridium difficile at the very early stages of infection. Therefore, *Romboutsia* plays a critical role in deciphering the link between gut microbiota and Clostridium difficile infection (58) and has been suspected to be depleted in patients with malnutrition or patients with intestinal polyps (59, 60). However, *Oscillibacter* and *Enterobacter*, which are relatively abundant in the patient group, are regarded as opportunistic pathogenic bacteria. Previous studies have reported that the decreased abundance of *Oscillibacter* genus may promote obesity and type 2 diabetes (61); however, its increased abundance has been demonstrated in major depressive disorder (62). As for *Enterobacter*, several studies have shown that it is closely associated with the development of many tumors, such as colorectal, liver, lung, and bladder cancers (63–66). Reduced abundance of taxa such as *Blautia* and *Romboutsia* is potentially detrimental to maintaining homeostasis and makes the body vulnerable to diseases. Meanwhile, the increased abundance of opportunistic pathogens such as *Oscillibacter* and *Enterobacter* is prone to induce various diseases. Undeniably, overproduction of GH and IGF-1 leads to complications of multiple systems, including mainly cardiomyopathy, ventilatory dysfunction, metabolic and endocrine complications, colonic polyps, and intestinal tumors (22, 67, 68), which may be related to the disturbance of the above-mentioned bacterial flora. However, the current research cannot accurately explain whether the disturbance of the bacterial flora is caused by the hypersecretion of GH/IGF-1.

By using shotgun metagenomics sequencing to describe the microbiota changes at the species level in GHPA, we found a higher abundance of Alistipes shahii species in the patients. *Alistipes* is a relatively new genus of bacteria isolated primarily from medical clinical samples, and although at a low rate compared to that of other genus members of the *Bacteroidetes* phylum, *Alistipes* is highly relevant in dysbiosis and diseases (69). Several studies have indicated that *Alistipes* is pathogenic in colorectal cancer and is associated with the mental signs of depression (70, 71). Because intestinal tumors are a common and serious complication of GHPA (68), we suppose that Alistipes shahii may play a positive role in the occurrence of intestinal tumors in the patients.

Previous studies have speculated that the association between microbiota and GH/IGF-1 axis is interactive. Yan et al. reported that gut microbiota and microbial metabolite short-chain fatty acids (SCFAs) produced by microbiota significantly increase IGF-1 production (2, 72). Jensen et al. demonstrate that GH excess may cause significant microbial differences in mice, indicating the effects of GH on the microbiota (16). In our work, we found a positive correlation between *Enterobacter* and preoperative level of IGF-1 in GHPA and further detected positive correlations between *Enterobacter* sp. *DC1* and ratio of change in fasting GH as well as ratio of change in nadir GH levels, without notable positive associations between IGF-1 and species of *Enterobacter* identified. Since the correlation analyses at genus and species levels were based on the 16S rRNA and metagenomics sequencing, respectively, the intrinsic bias due to unequal numbers of participants may interfere with our exploration. Nonetheless, positive correlations between species of *Enterobacter* and ratio of change in fasting as well as nadir GH level denoted the positive relationship between this taxon and GH level, thus conceivably affecting the downstream IGF-1 in a positive manner. Collectively, our findings indicated that *Enterobacter* may be positively correlated with the GH/IGF-1 axis. However, the mechanism underlying the interaction between *Enterobacter* and GH/IGF-1 axis remains unclear. Further, we used the FishTaco analysis, quantifying the contribution of taxa to observed functional shifts via permutation-based approach as reported by Manor and Borenstein (50), and found that two species of *Enterobacter* (*Enterobacter_sp_940_PEND* and *Enterobacter_sp_DC1*) attenuated pathways which significantly changed between GHPA and healthy controls, including glucose metabolism. Thus, we speculate that long-term effects of *Enterobacter* may cause glucose metabolism disorder. Patients with GHPA are prone to diabetes due to the excess secretion of GH (22); therefore, the connection between *Enterobacter* and GH may be synergistic.

In conclusion, by using 16S rRNA amplicon sequencing and metagenomics shotgun sequencing, we confirmed a decreased diverse microbiota in patients with GHPA, identified the increased levels of *Oscillibacter* and *Enterobacter* in the patients, and were first to propose that the *Enterobacter* genus may be strongly associated with GH/IGF-1 axis in GHPA. The results of the present study extend our insights into GHPA microbiome, which may clarify GHPA pathogenesis and facilitate the exploration of novel therapeutic targets based on microbiota manipulation. However, prospective studies are needed to further define the role of these microbiota in the development of GHPA.

## MATERIALS AND METHODS

### Clinical parameters, abbreviations, and formulas.

Clinical indices mentioned in this study included demographic features (age, gender, and body mass index), comorbidities (hypertension [HBP] and diabetes mellitus [DM]; diagnoses were based on 2017 American Heart Association [73] and 1999 World Health Organization criteria [74], respectively), tumor features (including the recurrence of tumor, tumor size, Knosp grading of tumor, a grading system to evaluate invasiveness of pituitary adenoma as well as granulation extent of tumor, a pathological index associated with aggressiveness and resistance to pharmacological therapy [75, 76]), laboratory tests (fasting plasma glucose [FPG], fasting GH, nadir GH, IGF-1, and IGF-1 index), and endpoints of management (biochemical remission was defined as an age-normalized serum IGF-1 value and a random GH of <1.0 ng/mL according to existing consensus (30), and we defined radiological remission as no recurrence or residual tumor on follow-up magnetic resonance imaging).

GH group and HC group are abbreviations of growth hormone-secreting pituitary adenoma group and healthy controls group, respectively. The recurrence of tumor denoted the status that the patient carried the recurrent tumor despite past treatment. Body mass index (BMI) was calculated by weight/(height)^2^, where weight is in kilograms and height is in meters. Tumor size was evaluated via the formula 1/2 × length × width × height, where all measurements are in millimeters. IGF-1 index was the ratio of the measured IGF-1 value to the upper limit of normal. Fasting GH refers to the GH level at the 0 point during oral glucose tolerance test (OGTT), while nadir GH is the nadir GH level during OGTT.

Clinical parameters were calculated as follows. Change in fasting GH = preoperation fasting GH – postoperation fasting GH. Change in nadir GH = preoperation nadir GH – postoperation nadir GH. Change in IGF-1 index = preoperation IGF-1 index – postoperation IGF-1 index. Ratio of change in fasting GH = (preoperation fasting GH – postoperation fasting GH)/preoperation fasting GH. Ratio of change in nadir GH = (preoperation nadir GH – postoperation nadir GH)/preoperation nadir GH. Ratio of change in IGF-1 index = (preoperation IGF-1 index – postoperation IGF-1 index)/preoperation IGF-1 index. Postoperative values were obtained 6 months after surgery.

### Participants and study design.

This study was approved by the local ethics committee of the Huashan Hospital (KY2015-256), Fudan University, and all participants gave informed consent. Patients with newly diagnosed GH-secreting pituitary adenoma (GHPA) were recruited from the Department of Neurosurgery from March 2018 to April 2019. Diagnostic criteria of GHPA were based on the endocrine society clinical practice guideline (30). To exclude the potential effect on microbiome analysis from potential confounding factors, exclusion criteria were (i) antibiotics and/or probiotics usage within 6 months, (ii) a history of gastrointestinal tumors and/or inflammatory diseases, and (iii) immunosuppressive drug usage within 6 months.

Clinical parameters were collected during the perioperative period and follow-up hospitalization on the sixth month after surgery. Clinical information included demographic features (age, gender, and body mass index), comorbidities (hypertension and diabetes mellitus), tumor features (recurrence, tumor size, Knosp grade, and dense/sparse granulation), laboratory tests (fasting plasma glucose, fasting and nadir growth hormone level during oral glucose tolerance test, insulin-like growth factor-1, and IGF-1 index), and endpoints of management (biochemical remission as well as radiological remission). Further, preoperation BMI, FPG, fasting and nadir GH, IGF-1, IGF-1 index, and their counterparts were compared 6 months after surgery.

Healthy controls with general demographics matched were selected from a large cohort established by the Department of General Surgery, Shanghai Tenth People’s Hospital, School of Medicine, Tongji University (77). The exclusion criteria mentioned above were also applied to the control group.

A total of 28 patients with GH-secreting adenoma and 67 healthy controls were included in this study, and their stool samples were submitted to 16S rRNA amplicon sequencing. Meanwhile, stool samples of 9 patients and 10 healthy controls were randomly selected and further analyzed by metagenomics sequencing. Detailed composition, biodiversity, taxonomic difference, and potential functional implication of the gut microbiota were delineated. Furthermore, the correlation between gut microbiome and clinical characteristics was analyzed. A classifier to discriminate GH-secreting adenoma from the general population was also constructed and validated.

### Biochemical measurements.

GH and IGF-1 were measured as described previously (78). Briefly, GH was measured by a two-site chemiluminescent immunometric assay (AutoDELFIA hGH, PerkinElmer Life and Analytical Sciences, Wallac Oy), with intraassay coefficient of variation (CV) of 5.3 to 6.5%, interassay CV of 5.7 to 6.2%, and sensitivity up to 0.01 μg/L (0.026 mU/L).

IGF-1 was measured with the Immulite 2000 solid-phase, enzyme-labeled chemiluminescent immunometric assay (Siemens Healthcare Diagnostic Products Limited, UK); normal age-appropriate ranges are as follows:1 to 6 years, 49 to 327 μg/L; 7 to 11 years, 57 to 551 μg/L; 12 to 13 years, 143 to 850 μg/L; 14 to 16 years: 220 to 996 μg/L; 17 to 18 years, 163 to 731 μg/L; 19 to 20 years, 127 to 483 μg/L; 21 to 35 years, 115 to 358 μg/L; 36 to 50 years, 94 to 284 μg/L; >50 years, 55 to 238 μg/L; intraassay CV, 2.3 to 3.5%; interassay CV, 7.0 to 7.1%; sensitivity, 20 μg/L. IGF-1 index = IGF-1/upper limit of normal range (ULN) (79).

Plasma glucose was measured by HITACHI 7600 Biochemical Analyzer (Tokyo, Japan).

### Collection and processing of feces samples.

Fecal samples of patients were collected preoperatively via Sarstedt feces collection containers (SARSTEDT, Nümbrecht, Germany) following the manufacturer’s instructions at the hospital, immediately frozen, transferred to our lab on ice, and immediately stored at −80°C until sample processing. Fecal samples were collected by the same staff. As for the healthy controls group, fecal samples were processed and completed during community gastrointestinal disorders screening according to the identical protocol as mentioned above.

### 16S rRNA gene amplicon sequencing.

DNA was extracted from each fecal sample using improved protocol based on the QIAamp Fast DNA stool minikit (Qiagen, Germany). In detail, 1 mL of InhibitEX buffer and a proper amount of glass beads (0.5 mm diameter, Qiagen) were added to each 200-mg sample of feces. The mixture was homogenized and beat with 60 Hz for 1 min twice with a homogeneous instrument (FASTPREP-24, Aosheng Biotech, China). Afterwards, the DNA purification was performed according to the manufacturer’s instructions.

The V3-V4 region of the bacteria 16S rRNA genes was amplified by PCR (95°C for 3 min, followed by 30 cycles at 98°C for 20s, 58°C for 15s, and 72°C for 20s and a final extension at 72°C for 5 min) using barcoded primers 341F 5′-CCTACGGGRSGCAGCAG-3′ and 806R 5′-GGACTACVVGGGTATCTAATC-3′. PCRs were performed in a 30 μL mixture containing 15 μL of 2× KAPA Library Amplification ReadyMix, 1 μL of each primer (10 μM), 50 ng of template DNA, and ddH_2_O. Negative controls consisting of empty sterile storage tubes were processed for DNA extraction and amplification using the same procedures and reagents used for the 95 samples. There was no detectable amplification in the negative controls. Amplicons were extracted from 2% agarose gels and purified using the AxyPrep DNA gel extraction kit (Axygen Biosciences, Union City, CA, USA) according to the manufacturer’s instructions and quantified using Qubit 2.0 (Invitrogen, USA). All quantified amplicons were pooled to equalize concentrations for sequencing using Illumina MiSeq/HiSeq (Illumina, Inc., CA, USA). The paired-end reads of 250 bp were overlapped on their 3 ends for concatenation into original longer tags by using PANDAseq (https://github.com/neufeld/pandaseq, version 2.9) (80). DNA extraction, library construction, and sequencing were conducted at Realbio Genomics Institute (Shanghai, China).

### Analysis of 16S rRNA gene amplicon sequencing data.

Assembled tags, trimmed of barcodes and primers, were further checked on their rest lengths and average base quality. 16S tags were restricted between 220 bp and 500 bp such that the average Phred score of bases was no worse than 20 (Q20) and no more than 3 ambiguous bases (N). The copy number of tags was enumerated and redundancy of repeated tags was removed. Only the tags with frequency more than 1, which tend to be more reliable, were clustered into OTUs, each of which had a representative tag. Operational taxonomic units (OTUs) were clustered with 97% similarity using UPARSE (http://drive5.com/uparse/) (81), and chimeric sequences were identified and removed using Usearch (version 7.0.1090) (82). Each representative tag was assigned to a taxon by RDP Classifier against the RDP database (http://rdp.cme.msu.edu/) (83) using confidence threshold of 0.8. OTU profiling table, and alpha diversity analyses were also achieved by python scripts of QIIME (version 1.9.1) (84).

Weighted UniFrac uses phylogenetic information to compare microbial community differences between samples, taking the abundance of sequences into account. The calculated results, taking into account the evolutionary distance between taxa, are used as an index to measure beta diversity. The larger the index, the greater the difference between samples. By clustering the results of UniFrac, samples with smaller UniFrac distance are clustered together, reflecting the similarity among samples. In the current study, we exploited QIIME to perform weighted UniFrac analysis, with detailed codes provided in github repository (https://github.com/xxhym/bioinformatic_codes), and original script can be found on the website http://qiime.org/scripts/beta_diversity.html. The results were presented via principal coordinates analysis (PCoA) and nonmetric multidimensional scaling (NMDS) analysis. To depict the variance between different groups, Adonis test together with principal coordinates analysis (PCoA) were exploited. In addition, multiresponse permutation procedure (MRPP), a mathematic form to present difference in weighted UniFrac (85, 86), was utilized to collaborate with NMDS analysis to better illustrate the intergroup difference.

To acquire differential taxa, LEfSe analysis and intergroup rank-sum test analysis were conducted. LEfSe was conducted by means of nonparametric Kruskal-Wallis test, and it used linear discriminant analysis (LDA) to estimate the influence of the abundance of each component (taxon) on the difference effect, thus illustrating the communities or taxa with significant difference influence on sample division (87). The detailed code is provided in our github repository (https://github.com/xxhym/bioinformatic_codes), and the analysis can also be achieved either online (http://huttenhower.sph.harvard.edu/galaxy/root/index) or via local software (https://toolshed.g2.bx.psu.edu/). On the other hand, the rank-sum test was used to analyze the significant differences between different groups to identify the species that had significant effects on the division between groups. In this analysis, the Wilcox test function of the stats package in R was used for the difference analysis between two groups, and the Kruskal test function of the stats package in R was used for the difference analysis among more than two groups.

Thereafter, using phylotype at genus level and clinical indices of GH patients, the correlations between the two components are calculated to unfold important internal relationships, graphically shown through a heatmap generated through corrplot package in R.

### Metagenomics shotgun sequencing.

DNA was extracted from each fecal sample using improved protocol based on the manual of QIAamp Fast DNA stool minikit (Qiagen, Germany). In detail, 1 mL of InhibitEX buffer and a proper amount of glass beads (0.5 mm diameter, Qiagen) were added to each 200-mg sample of feces. The mixture was homogenized and beat with 60 Hz for 1 min twice with a homogeneous instrument (FASTPREP-24, Aosheng Biotech, China). Afterwards, the DNA purification was performed according to the manufacturer’s instructions, the DNA concentration was measured with a NanoDrop (Thermo Scientific) and Qubit 2.0 (Invitrogen, USA), and the molecular size was estimated by agarose gel electrophoresis.

Following the Illumina TruSeq DNA Sample Prep v2 guide (Illumina, Inc., San Diego, CA, USA), we constructed the DNA libraries with approximately 500 bp insert sizes for each sample. The quality of all libraries was evaluated using an Agilent 2100 bioanalyzer (Agilent Technologies, Wokingham, UK) and the Agilent 2100 DNA 1000 kit. All samples were subject to 150 bp paired-end sequencing on an Hiseq X-10 platform (Illumina, Inc., San Diego, CA, USA).

Illumina raw reads were screened according to the following criteria: (i) adaptor contamination reads were removed, (ii) reads containing more than three ambiguous N bases were removed, (iii) reads containing low-quality (Q < 20) bases were trimmed, and (iv) reads containing less than 60% of high-quality bases (Phred score ≥ 20) were deleted. Then, clean reads were subjected to bacterial genomes from the National Center for Biotechnology Information GenBank with SOAPaligner (version 2.21) (88) and reads that mapped to the host genome were abandoned. The subsequent reads were selected for further analysis.

Clean reads were aligned to the NCBI GenBank database (https://ncbi.nlm.nih.gov/genbank/) for the detection of known bacteria, fungi, viruses, and archaea by SOAPaligner 2.21. Then, the aligned reads were classified as kingdom, phylum, class, order, family, genus, and species to count classification and abundance, and taxonomic relative abundance profile at different levels was generated.

Preprocessed reads were assembled by SOAPdenovo (version 1.05) (89). During the assembly process, the assembled scaffolds were split into contigs at ambiguous Ns and contigs no less than 500 bp were kept for further analysis. The k-mers with maximum *N*_50_ were selected as the final assembly results. Software MetaGeneMark (http://exon.gatech.edu/GeneMark/meta_gmhmmp.cgi) (90) was used to predict open reading frames (ORFs) in the assembled scaffolds, and predicted ORFs less than 100 bp were removed.

The nonredundant gene catalog set was constructed by pairwise comparison of predicted ORFs (gene length > 100 bp) using CD-HIT (version 4.5.7) (91). Two sequences with coverage of ≥90% and identity of ≥95% were considered redundant, and the longer one was regarded as the representative. The final nonredundant gene catalog contained 885,596 ORFs with an average length of 818.90 bp.

Clean reads were aligned to the genes in the nonredundant catalogue by using SOAPaligner. The calculation formula of gene abundance used was from the study by Qin et al. (92).

Using BLAST (version 2.2.28+) (93), the nonredundant genes were annotated against the KEGG (Kyoto Encyclopedia of Genes and Genomes) database. When assembled protein sequence was similar (score of ≥ 60 and E value of <1e–5) to a protein sequence in the database, the assembled protein was considered to play the same role as the protein in the database. We accumulated the relative abundance of all orthologous genes to generate the relative abundance of each KO.

### Analysis of metagenomics shotgun sequencing.

According to the abundance of samples, the Bray-Curtis distance between samples was calculated and the hierarchical clustering was carried out to analyze the distance relationship between samples and whether there were outliers.

Afterwards, based on the abundance of species, the *P* value of each taxon at species level was calculated by Wilcox rank-sum test. Then, the species with significant difference between the two groups were screened out according to the *P* value and exhibited in the form of boxplots, with the general threshold of a *P* value of ≤0.05. Meanwhile, LEfSe analysis was initiated to probe microbial biomarkers and reveal genomic characteristics. Similar to LEfSe analysis with 16S rRNA, we used the nonparametric factorial Kruskal-Wallis (KW) sum-rank test to detect taxa with significantly different abundance between the two groups, and thereafter linear discriminant analysis (LDA) was used to estimate relative influence resulting from abundance of each component (species) on the total intragroup difference (87), thus locating species fundamental in respect to sample division.

Then, we combined the abundance of detected genes and the corresponding annotation from KEGG database to describe relative abundance of comprehensive functional elements at level 1 and level 2 of each sample in the form of barplots.

Thereafter, integrating taxa at species level and clinical parameters of GH patients, the spearman correlations between the two components were calculated to unveil vital internal relationships, presented as a heatmap generated through corrplot package in R.

Last, but not least, the FishTaco analysis (50), based on permutation-based approach to link species to gene function/metabolic pathways, was carried out in participants with metagenomics. Therefore, driving factors responsible for the functional changes and their corresponding contributions to the alterations were attained, probing into the possible mechanism of changes in gene function/metabolism pathway caused by species.

### Statistical analysis.

Statistical analysis of the clinical metadata was conducted in SPSS. Continuous data were described as mean ± standard deviation (interquartile range for nonnormalized data), and the count data were described as count and proportion. Paired *t* tests were used to assess the differences between normally distributed continuous variables, and Mann-Whitney U tests were used with variables that failed the normality test. The false-discovery rate (FDR) algorithm was used for multiple comparisons to control the chance of generating unwanted false positives.

### Data availability.

All bioinformatic codes are available on Github repository (https://github.com/xxhym/bioinformatic_codes). 16S rRNA amplicon sequencing resources are available on BioProject ID PRJNA743650, and metagenomics sequencing resources are available on BioProject ID PRJNA759070.
