# Supplementary material for: Characteristics of Gut Microbiota in Patients with GH-Secreting Pituitary Adenoma
Source: Microbiol Spectr. 2022 Jan 12;10(1):e00425-21. doi: 10.1128/spectrum.00425-21 (PMC8754134; doi:10.1128/spectrum.00425-21)

**SUPPLEMENTARY FIG 1 Gut microbial alterations of biodiversity in GH group**

(A) Rarefaction curves between number of samples and detected OTUs. The estimated OTUs richness basically approached saturation in all samples. (B) Beta diversity was calculated using weighted UniFrac by Anosim test, inter-group variance was not significant. (C) There was no significant alteration in alpha diversity of microbiota between two groups when alpha diversity was estimated by chao1 diversity index, goods coverage diversity index, observed species diversity index or PD whole tree diversity index. GH, growth hormone secreting pituitary adenoma; HC, healthy control; OTUs, Operational Taxonomy Units.

**SUPPLEMENTARY FIG 2 Taxonomic profiles and differential taxa of both groups**

(A) The histogram exhibited total composition of bacterial community at the genus level in all participants in 16S rRNA sequencing part. Only the 20 main genus in both groups were shown for clarity. (B) The histograms demonstrated the relative abundance of phylum *Bacteroidetes* as well as *Firmicutes* of GH groups and HC groups in 16S rRNA sequencing part, while the boxplot represented the corresponding *Firmicutes/Bacteroidetes* ratio between two groups in 16S rRNA sequencing part. (C) The histograms demonstrated the relative abundance of phylum *Bacteroidetes* along with *Firmicutes* of two groups in metagenomics sequencing, while the boxplot and violin plot represented the corresponding *Firmicutes/Bacteroidetes* ratio and abundance of *Oscillibacter* between two groups in metagenomics sequencing. (D) Boxplots illustrated differential taxa at genus level in patients and healthy individuals detected by rank-sum test in metagenomics sequencing part, with two different colors representing different sets of samples while the short bar and the plus sign indicating the median and the mean respectively.

Only the 20 most abundant genus in each group are shown for clarity. GH, growth hormone secreting pituitary adenoma; HC, healthy control; p_ and g_ in figure b and c were short for phylum and genus respectively; dotted line and dashed line in violin plot of figure c represented quartiles and median respectively.

**SUPPLEMENTARY FIG 3 Functional profiles of participants in this study**

(A) Classification of KEGG annotation results of gene sets detected by metagenomics sequencing were displayed as a histogram, with a noticeable proportion of the overall genes in both groups involved in metabolism. The vertical axis was the name of KEGG metabolic pathway, meanwhile the abscissa referred to the relative proportion of the number of genes annotated to specific pathway in the total number of genes. The figure legend on right side stands for the classification of the pathways at Level 1. The annotation information of Level 1 and Level 2 obtained from KEGG database were then combined with corresponding KOs abundance to obtain relative abundance of pathways at level 1 (B) as well as level 2 (C) in each group. The ordinates stood for the relative abundance of Level 1 (B) and 2 (C) pathways of KEGG, with specific illustration of each pathways listed on the right side. KEGG: Kyoto Encyclopedia of Genes and Genomes; GH, growth hormone secreting pituitary adenoma; HC, healthy control.

**SUPPLEMENTARY FIG 4 Fishtaco analysis of differential ko detected in metagenomics sequencing**

Fishtaco analysis was conducted to determine which species or populations of species were

correlated to differential gene function/metabolic pathways in our microbial samples, acquiring the driving factors of the functional changes as well as their specific contributions. In our figure, rank sum scores were shown on the horizontal axis, and differential functional modules were shown on the vertical axis. As shown, the driving factors of each differential functional transformation were divided into four parts and represented by a two-direction histogram. In the GH group, microbes that promoted the corresponding ko were presented on the upper right side and microbes that had inhibitive effect were presented on the upper left side. As for the HC group, microbes that promoted the corresponding ko were presented on the lower right side and microbes that had inhibitive effect were presented on the lower left side. Different colored bars symbolized the corresponding species, and the longer the bar, the greater the driving or inhibition of the corresponding function by the species. And our results indicated the prominent role of *Alistipes shahii* as a major contributor in respect to amino acids metabolism, carbohydrate metabolism and energy metabolism, with species of *Enterobacter* genus being the suppressive factors in amino acids metabolism, carbohydrate metabolism and energy metabolism. ko: reference pathway of KEGG database.

SUPPLEMENTARY FIG 1

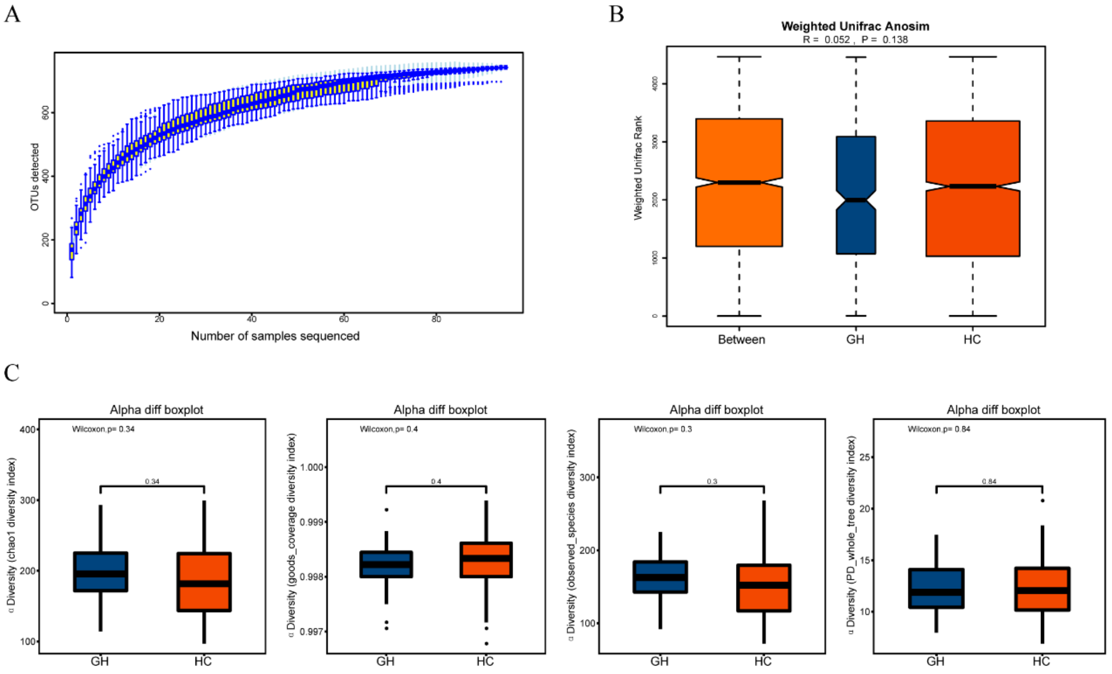

SUPPLEMENTARY FIG 2

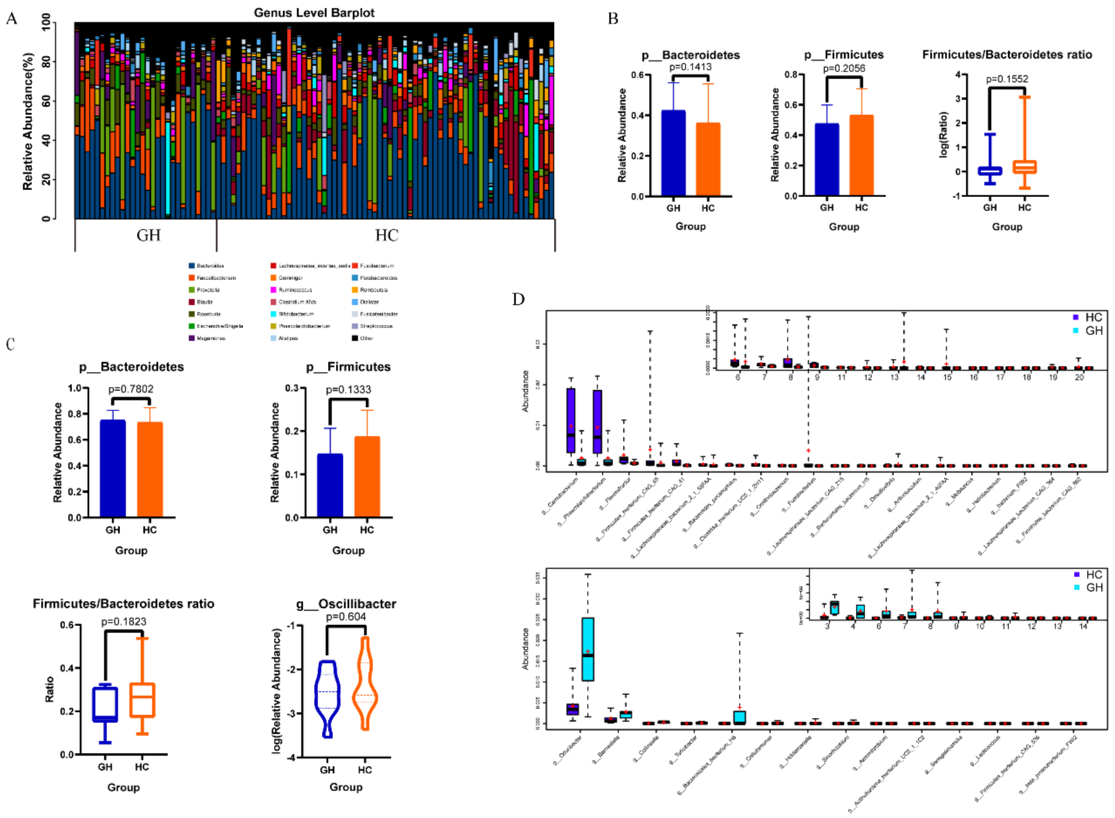

# SUPPLEMENTARY FIG 3

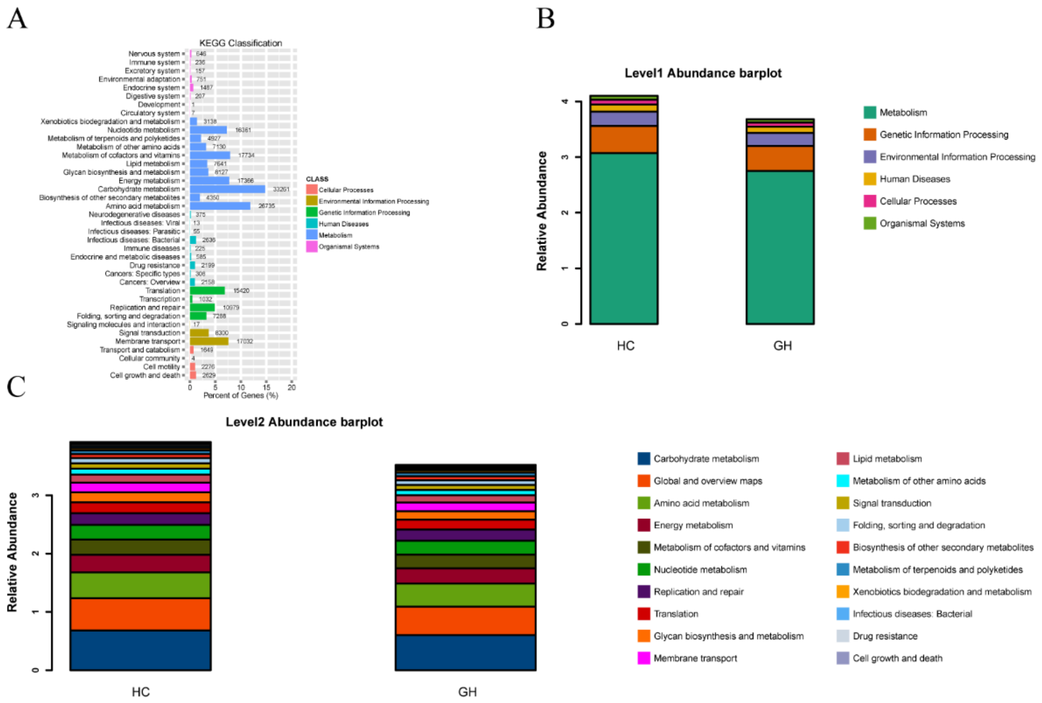

# SUPPLEMENTARY FIG 4

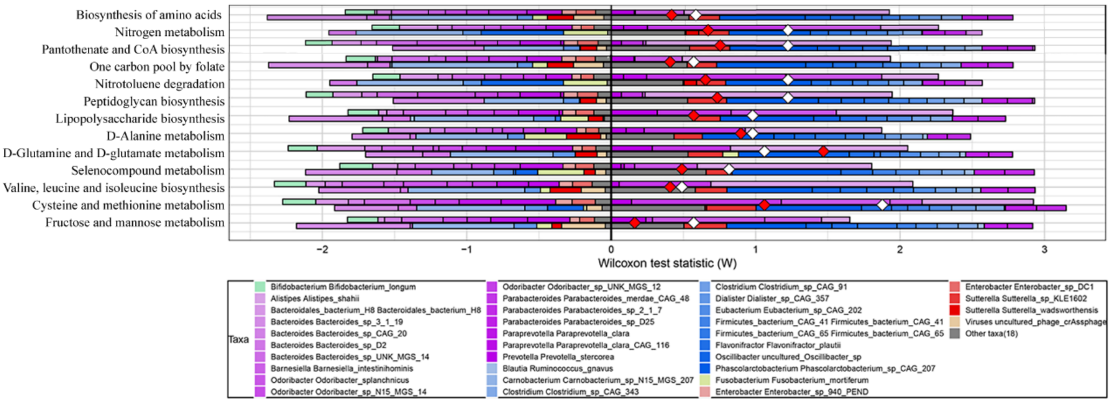

Supplement: SUPPLEMENTAL FILE 3 — Supplemental material. Download SPECTRUM00425-21_Supp_1_seq11.pdf, PDF file, 0.9 MB [file spectrum00425-21_supp_1_seq11.pdf]
